# VTSNN: a virtual temporal spiking neural network

**DOI:** 10.3389/fnins.2023.1091097

**Published:** 2023-05-23

**Authors:** Xue-Rui Qiu, Zhao-Rui Wang, Zheng Luan, Rui-Jie Zhu, Xiao Wu, Ma-Lu Zhang, Liang-Jian Deng

**Affiliations:** ^1^School of Optoelectronic Science and Engineering, University of Electronic Science and Technology of China, Chengdu, China; ^2^School of Public Affairs and Administration, University of Electronic Science and Technology of China, Chengdu, China; ^3^School of Mathematical Sciences, University of Electronic Science and Technology of China, Chengdu, China; ^4^School of Computer Science and Engineering, University of Electronic Science and Technology of China, Chengdu, China

**Keywords:** spiking neural networks, undistorted weighted-encoding/decoding, neuromorphic circuits, Independent-Temporal Backpropagation, biologically-inspired artificial intelligence

## Abstract

Spiking neural networks (SNNs) have recently demonstrated outstanding performance in a variety of high-level tasks, such as image classification. However, advancements in the field of low-level assignments, such as image reconstruction, are rare. This may be due to the lack of promising image encoding techniques and corresponding neuromorphic devices designed specifically for SNN-based low-level vision problems. This paper begins by proposing a simple yet effective undistorted weighted-encoding-decoding technique, which primarily consists of an Undistorted Weighted-Encoding (UWE) and an Undistorted Weighted-Decoding (UWD). The former aims to convert a gray image into spike sequences for effective SNN learning, while the latter converts spike sequences back into images. Then, we design a new SNN training strategy, known as Independent-Temporal Backpropagation (ITBP) to avoid complex loss propagation in spatial and temporal dimensions, and experiments show that ITBP is superior to Spatio-Temporal Backpropagation (STBP). Finally, a so-called Virtual Temporal SNN (VTSNN) is formulated by incorporating the above-mentioned approaches into U-net network architecture, fully utilizing the potent multiscale representation capability. Experimental results on several commonly used datasets such as MNIST, F-MNIST, and CIFAR10 demonstrate that the proposed method produces competitive noise-removal performance extremely which is superior to the existing work. Compared to ANN with the same architecture, VTSNN has a greater chance of achieving superiority while consuming ~1/274 of the energy. Specifically, using the given encoding-decoding strategy, a simple neuromorphic circuit could be easily constructed to maximize this low-carbon strategy.

## 1. Introduction

Spiking Neural Networks (SNNs) are artificial neural networks of the “third generation” that closely resemble natural neural networks (Maass, 1997). Since biological motion processing depends on temporal information and gains superb performances (Saygin, 2007). Researchers attempt to use SNN to convert spatial complication to temporal complication. Since the information is transmitted in the form of spikes. It also has a lower carbon footprint (Roy et al., 2019) and superior robustness (Sironi et al., 2018). SpikeProp (Bohte et al., 2002) initially updated weights using SNN with backpropagation and supervised learning. Few studies are devoted to low-level image tasks with supporting neuromorphic chips, and the majority of SNNs are currently focused on classification (Xing et al., 2020; Fang et al., 2021; Zheng et al., 2021).

In addition, the vast majority of SNNs designed for low-level tasks require specialized hardware such as event-based cameras (Zhang et al., 2021; Zhu et al., 2022). This requirement substantially raises the bar for usage. Pioneers in this area introduced a novel SNN, requiring no specialized hardware (Comşa et al., 2021). Their performance, however, is not ideal, and our work will improve it. Since 2002 (Bohte et al., 2002), the surrogate gradient has been commonly employed for backpropagation in SNN, then Neftci et al. (2019) introduced Backpropagation Through Time (BPTT) to this area. Besides, Deng et al. (2022) assert standard direct training by utilizing a formula to distinguish it from ANN-SNN conversion. Also, hybrid ANN-SNN conversion requires additional time steps and must be shadow trained exclusively (Eshraghian et al., 2021). Inspired by related studies (Werbos, 1990), Spatio-Temporal Backpropagation (STBP) is introduced.

Since 2002 (Bohte et al., 2002), the surrogate gradient has been commonly employed for backpropagation in SNN, then Neftci et al. (2019) introduced Backpropagation Through Time (BPTT) to this area. Besides, Deng et al. (2022) assert standard direct training by utilizing a formula to distinguish it from ANN-SNN conversion. Also, hybrid ANN-SNN conversion requires additional time steps and must be shadow trained exclusively (Eshraghian et al., 2021). Inspired by related studies (Werbos, 1990), Spatio-Temporal Backpropagation (STBP) is introduced.

Other related approaches include Temporal Spike Sequence Learning via Backpropagation (TSSL-BP) (Zhang and Li, 2020) but only appropriate for the classification task. For the low-level denoising assignment in this work, STBP performs worse (Comşa et al., 2021) than our Independent-Temporal Backpropagation (ITBP).

Rate coding, temporal coding, delta modulation, and direct coding are four common encodingmethods. Among them, delta modulation and rate coding lose pixel location information (Kim et al., 2022). Direct coding can maintain location information, but it cannot be analyzed quantitatively (Jin et al., 2022). Weighted phase spiking coding (a type of temporal coding) employs the binary encoding concept (Kim et al., 2018). But it is also distorted and requires a normalization trick. Comşa et al. (2021) employed a latency coding method called time-to-first-spike (TTFS), inspired by biological vision (Hubel and Wiesel, 1962), to represent pixel brightness. TTFS cannot guarantee undistorted results, needs more time steps, and performs worse than ours. The classification task does not generate images; consequently, there are few decoding methods for low-level tasks such as reconstruction. Membrane Potential Decoding (MPD) (Kamata et al., 2022) is, to the best of our knowledge, the only appropriate decoding method. However, MPD generates floating results, necessitating the inclusion of a surrogate function. Prior to our work, there was no symmetric and undistorted SNN encoding-decoding method. Rate coding, temporal coding, delta modulation, and direct coding are four common methods of encoding. Among them, delta modulation and rate coding lose pixel location information (Kim et al., 2022). Direct coding can maintain location information, but it cannot be analyzed quantitatively (Jin et al., 2022). Weighted phase spiking coding (a type of temporal coding) employs the binary encoding concept (Kim et al., 2018). But it is also distorted and requires a normalization trick. Comşa et al. (2021) employed a latency coding method called time-to-first-spike (TTFS), inspired by biological vision (Hubel and Wiesel, 1962), to represent pixel brightness. TTFS cannot guarantee undistorted results, needs more time steps, and performs worse than ours. The classification task does not generate images; consequently, there are few decoding methods for low-level tasks such as reconstruction. Membrane Potential Decoding (MPD) (Kamata et al., 2022) is, to the best of our knowledge, the only appropriate decoding method. However, MPD generates floating results, necessitating the inclusion of a surrogate function. Prior to our work, there was no symmetric and undistorted SNN encoding-decoding method.

This paper here presents a Virtual Temporal Spiking Neural Network (VTSNN) for image reconstruction. VTSNN is based on a modified U-net (Ronneberger et al., 2015) which is a classical architecture. There are many works that apply U-shape architecture to do image reconstruction tasks such as image denoising and achieving promising results (Yue et al., 2020; Cheng et al., 2021; Zamir et al., 2021; Wang et al., 2022). Alternatively, we propose an Undistorted Weighted-Encoding-Decoding method for converting an arbitrary image into binary data (0/1) in order to efficiently encode image data. We also demonstrate that this encoding-decoding procedure can be performed by simple neuromorphic circuits, thereby increasing its effectiveness. The schematic diagram of the circuits consists of ADC and DAC. Additionally, we propose a novel backpropagation technique called Independent-Temporal Backpropagation (ITBP) to avoid the inefficiency of Spatio-Temporal Backpropagation (STBP) (Wu et al., 2018). The main contributions of this paper can be summarized as follows:

- We propose, to the best of our knowledge, the first symmetric and undistorted encoding-decoding approach with high efficiency for fully spiking SNN-based image reconstruction tasks that can be implemented using simple neuromorphic circuits. This raises the prospect of low-level tasks being applied to neuromorphic devices.
- First, we introduce a virtual temporal SNN. This suggests that even without temporal information, SNN can be used to achieve competitive performance. A novel backpropagation for direct training, called ITBP, is also proposed for the designed encoding-decoding technique to improve effectiveness.
- Experimental results on a variety of datasets are often superior to the current SNN-based approach (Comşa et al., 2021) while superior to same-architecture ANN in some cases. In addition, VTSNN uses roughly 1/274 of the energy of ANN-based methods.

## 2. Method

Based on our analysis, the application of SNN and its neuromorphic devices is almost limited to the classification task. Consequently, we intend to investigate SNN's capabilities for low-level image tasks, such as reconstruction. In the meantime, popular input encoding methods have numerous shortcomings, including redundant time steps and information distortion. Moreover, studies on output decoding are quite rare. Therefore, we propose a novel symmetric and undistorted encoding-decoding method to fill the above gaps. Currently, researchers generally use STBP for the low-level SNN task (Comşa et al., 2021), which allows information to propagate in both temporal and spatial domains. Therefore, we present a new backpropagation that only permits information to propagate via the spatial domain. This backpropagation can improve the effectiveness and gain better performance. In addition, we want to use simple neuromorphic circuits to demonstrate the feasibility of our encoding-decoding method. With undistorted and symmetric encoding/decoding, simpler and more effective backpropagation, and fewer time steps, we aim to achieve competitive performance in low-level image reconstructing.

### 2.1. Preliminary

#### 2.1.1. Spiking neurons

Since 1907 (Lapique, 1907), qualitative scientific study has been conducted on the membrane voltage of neurons. Compared to the many-variable and intricate H-H model (Hodgkin and Huxley, 1952), the integrate-and-fire (IF) neuron model and leaky-integrate-and-fire (LIF) neuron model have a significantly reduced computational demand and are commonly recognized as the simplest models among all popular neuron models while retaining biological interpretability (Burkitt, 2006). The spiking neuron model is characterized by the following differential equation (Gerstner et al., 2014):


(1) $$\begin{array}{l} \tau \frac{du\left(t\right)}{dt}=-u\left(t\right)+x\left(t\right) \end{array}$$

Where *u*(*t*) represents the membrane potential of the neuron at time step *t*, *x*(*t*) represents the input from the presynaptic neurons, and τ is a time constant. What's more, spikes will fire if *u*(*t*) exceeds the threshold *V*_*th*_. The spiking neuron models can be described explicitly iteratively to improve computational traceability.


(2) $$x_{t+1,n}^{i}=\Sigma_{j}w_{n}^{j}o_{t+1,n-1}^{j}$$

(3) $$u_{t+1,n}^{i}=u_{t,n}^{i}\;g(o_{t,n}^{i})+x_{t+1,n}^{i}$$

(4) $$o_{t+1,n}^{i}=h(u_{t+1,n}^{i}-V_{th})$$

Here, *t* and *n*, respectively, represent the indices of the time step and *n*-th layer, and *o*^*j*^ is its binary output of *j*-th neuron. Furthermore, *w*^*j*^ is the synaptic weight from *j*-th neuron to *i*-th neuron, and by altering the way that *w*^*j*^ is linked, we can implement convolutional layers, fully connected layers, etc. To be more precise, the spiking neurons become the IF neuron if *g*(*x*) = τ and the LIF neuron if $g\left(x\right)=\tau e^{-\frac{x}{\tau }}$. Since *h*(·) represents the Heaviside function and Equation (**4**) is non-differentiable. The following derivatives of the surrogate function can be used for approximation.


(5) $$\begin{array}{l} \frac{\partial o_{t+1,n}^{i}}{\partial u_{t+1,n}^{i}}=\frac{1}{1+{\left(\pi x_{t+1,n}^{i}\right)}^{2}} \end{array}$$

**Figure 1:**
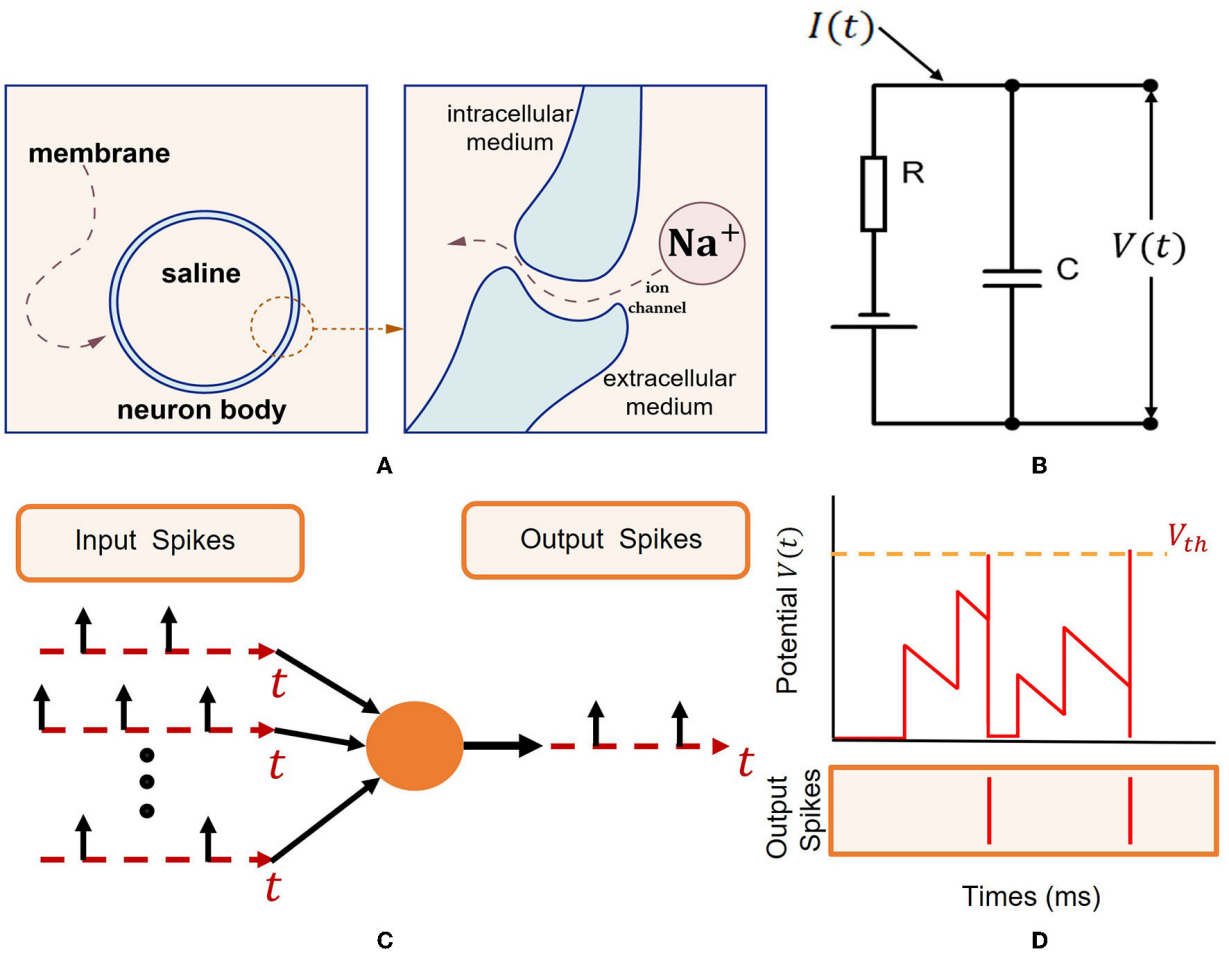
Spiking neuron model (Eshraghian et al., 2021). **(A)** Intracellular and extracellular mediums are divided by an isolating bilipid membrane. Gated ion channels allow ions such as *Na*^+^ to diffuse through the membrane. **(B)** Capacitive membrane and resistive ion channels constitute a resistor-capacitance circuit. A spike is generated when the membrane potential exceeds a threshold *V*_*th*_. **(C)** Via the dendritic tree, input spikes generated by *I* are transmitted to the neuron body. Sufficient excitation will cause output spike emission. **(D)** Simulation depicting the membrane potential *V*(*t*) reaching *V*_*th*_, resulting in output spikes.

The working schematic of spiking neurons is shown in Figure 1 (Eshraghian et al., 2021).

#### 2.1.2. Tensor multiplication

In Section 2.4, a transform pair for tensors are used to describe the decoding process. To better understand that process, here we first give some preliminary tensor definitions. A tensor with *N* dimensions is defined as $P\in {\mathbb{R}}^{I_{1}\times I_{2}\times \cdots \times I_{N}}$. Elements of P are denoted as *p*_*i*_1_, *i*_2_, ⋯ , *i*_*N*__, where 1 ≤ *i*_*n*_ ≤ *I*_*N*_. The *n*-mode unfolding vectors of tensor P are the *I*_*n*_-dimensional vectors obtained from P by changing index *i*_*n*_ while keeping the other indices fixed. The *n*-mode unfolding matrix $P_{\left(n\right)}\in {\mathbb{R}}^{I_{n}\times I_{2}I_{3}\cdots I_{n-1}I_{n+1}\cdots I_{N}}$ is defined by arranging all the *n*-mode vectors as the columns of the matrix (Kolda, 2006). The *n*-mode product of the tensor $P\in {\mathbb{R}}^{I_{1}\times I_{2}\times \cdots \times I_{N}}$ with the matrix $B\in {\mathbb{R}}^{J_{n}\times I_{n}}$, denoted by $P\times_{n}B$, is an *N*-dimensional tensor $Q\in {\mathbb{R}}^{I_{1}\times I_{2}\times \cdots \times J_{n}\cdots \times I_{N}}$. Hence, we have the following transform pair that will be used later in the image decoding process.


(6) $$\begin{array}{l} Q=P\times_{n}B\Leftrightarrow Q_{\left(n\right)}=BP_{\left(n\right)} \end{array}$$

### 2.2. Virtual temporal SNN

In this section, we propose and describe the concept of Virtual Temporal SNN (VTSNN):

*VTSNN is an abstract SNN definition that uses raw static data to generate spiking sequences (0/1) as network input, and the sequences are virtually ordered in the temporal domain*.

Specifically, VTSNN holds the following fundamental:

*The raw static data consists of non-temporal information and will be transformed into ordered sequences (a static encoding process), such as the operation of event-based hardware, rate coding, direct coding, etc*.

To realize the VTSNN, the crucial factors are to carefully design the corresponding encoding and decoding strategies which will be illustrated in detail.

### 2.3. Encoding

#### 2.3.1. Rethinking time-to-first-spike encoding (TTFS)

Previous study (Comşa et al., 2021) has applied a TTFS encoder to encode more salient information as earlier spikes and gained good results in reconstruction tasks. This encoding method is inspired by the idea of a rapid information process with spiking data (Thorpe et al., 2001). Here $r_{t}^{i,j}$ is the response of a pixel of an image at time step *t*. Equation (7) shows the calculation of $r_{t}^{i,j}$ for TTFS.


(7) $$r_{t}^{i,j}=\{\begin{array}{ll} 1 & t=(\frac{max(x)-x^{i,j}}{max(x)})T\\ 0 & \text{otherwise} \end{array}$$

In terms of $r_{t}^{i,j}$, after obtaining it, spike sequences are generated using the same methods as Algorithm 1 in this paper. There are two obvious disadvantages of TTFS.

- TTFS is distorted, which means not being capable of restoring information after coding, if solely uses a function to approach it.

**Algorithm 1:**
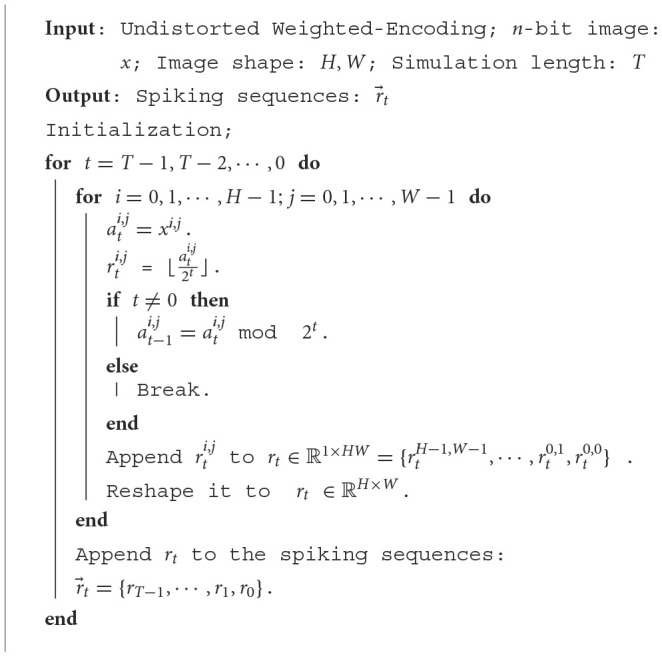
UWE algorithm for *n*-bit image.

#### 2.3.2. Undistorted weighted-encoding (UWE)

**Figure 2:**
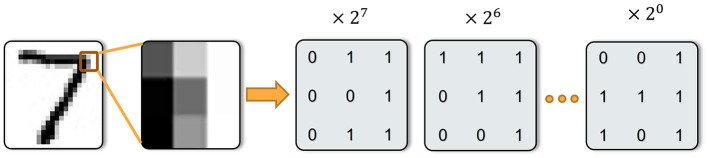
A toy example of our encoding. Here we demo the UWE with nine pixels as examples. For each pixel, the grayscale image was transferred into the eight-bit spike sequences and each bit was represented by a time step.

In this section, we propose the so-called Undistorted Weighted-Encoding (UWE) to encode the input images into spike sequences, as opposed to distorted encoders such as the time-to-first-spike (TTFS) encoder. Specifically, UWE can encode *n*-bit image [0, 2^*n*^−1] theoretically and a toy example of our coding is shown in Figure 2. In what follows, Algorithm 1 illustrates the process of encoding an image.

For simplicity, we set *n* = 8^1^ in our work, since the inputs are 8-bit image [0, 255]. Thus, we use 8-bit UWE in this work. Especially, in Algorithm 1, *x*^*i, j*^ is a pixel in the image *x*. After input encoding process, *x* is transferred into the spiking sequences ${\vec{r}}_{t}\in {\mathbb{R}}^{T\times H\times W}$and $r_{t}\in {\mathbb{R}}^{H\times W}$ accumulates information for each time step. Additionally, UWE is capable to be easily integrated with a neuromorphic chip which is introduced in the discussion part. This means *n*-bit image can be transferred into spiking sequences by the neuromorphic SAR ADC circuits (discussed in Section 4.4) without any floating arithmetic.

### 2.4. Decoding

#### 2.4.1. Rethinking membrane potential decoding (MPD)

The decoding method that (Kamata et al., 2022) uses for reconstruction tasks is categorized as MPD. Actually, MPD is similar to our Undistorted Weighted-Decoding (UWD) to some extent. This method, like UWD, applies a weight series to encode. However, the weight values of MPD are from 2 to 0.8 and it calls a float artificial neuron (*tanh* function) before returning outputs. This means the *n*-bit decoding matrix *A* in UWD is adjusted to Θ = {θ^*T*−1^, θ^*T*−2^, ⋯ , θ^0^} and θ = 0.8, then a *tanh* function is used to get the real-valued reconstructed image $\hat{Y}$. The mechanism of UWD will be introduced in the next section. Furthermore, there is a noticeable disadvantage: MPD will induct floating arithmetic, which is unfriendly to neuromorphic chips.

#### 2.4.2. Undistorted weighted-decoding

To overcome the disadvantages of existing decoders, we also present an Undistorted Weighted-Decoding (UWD) to decode the output spiking sequences ô_*t*_ ∈ ℝ^*H*×*W*^ (*t* = *T*−1, *T*−2, ⋯ , 0) into the final image $\hat{Y}$. This decoding process is actually a symmetric process of UWE, which means UWD will transform the spiking sequences into a *n*-bit image. According to the preliminary, we use the output spiking sequences ô_*t*_ (*t* = *T*−1, *T*−2, ⋯ , 0) to build a tensor $\hat{O}\in {\mathbb{R}}^{T\times H\times W}$. Then, we define a *n*-bit decoding matrix *A*∈ℝ^1 × *T*^ = {2^*T*−1^, 2^*T*−2^, ⋯ , 2^0^}. Similar to UWE, we also set *T* = 8 in the decoding process. In Section 2.1.2, we have already introduced tensor multiplication. The final decoding process can be described by the following formula:


(8) $$\begin{array}{l} \hat{Y}=\hat{O}\times_{n}A\Leftrightarrow {\hat{Y}}_{\left(3\right)}=A{\hat{O}}_{\left(3\right)} \end{array}$$

Where ${\hat{O}}_{\left(3\right)}\in {\mathbb{R}}^{T\times HW}$ is the 3-mode unfolding matrix of $\hat{O}$ while ${\hat{Y}}_{\left(3\right)}\in {\mathbb{R}}^{1\times HW}$ is the 3-mode unfolding matrix of $\hat{Y}\in {\mathbb{R}}^{H\times W\times 1}$. Hence, from the knowledge of tensor and transform pair effectively introduced in the preliminary part (Equation 6), we can get the final output image $\hat{Y}$. As the parallel inverse process of UWE, the decoding method can be realized by the neuromorphic chip we discussed later as well. The neuromorphic DAC circuits (discussed in Section 4.4) can convert spiking sequences to a real-valued reconstructed image without the use of floating-point arithmetic.

### 2.5. Spiking neural network architecture

As an abstract and flexible concept, VTSNN can be applied to various types of network architectures. In this work, our VTSNN is embedded in a shallow U-net architecture, named U-VTSNN. Because light U-net can extract features from images relatively efficiently. Additionally, unlike current SNNs for low-level image tasks whose data flow may contain floating numbers (Zhu et al., 2022), the U-VTSNN is a fully spiking neural network where all modules are built with SNN and all synapse operations are completed by spiking neurons (Kamata et al., 2022). In addition, U-VTSNN is a fully convolutional network while the biases of all convolutional layers are set to 0.

**Figure 3:**
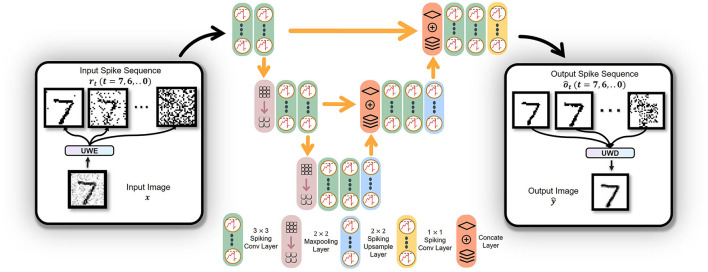
Architecture of the proposed fully spiking neural network with eight-bit as an example. UWE generates sequences from an input image. The sequences are fed into U-VTSNN. UWD generates images from operated sequences and finishes a complete noise-removal process. Additionally, the type and size of different layers are clearly shown above.

At the beginning of our image noise removal task, the image is transformed into spike sequences, which means a 1 × *H*×*W* tensor is fed into VTSNN and transformed as the size of *T*×*H*×*W* via UWE, followed by U-VTSNN. The details of the internal blocks are clearly shown in Figure 3. After all intermediate operations, the last block of U-VTSNN will output spiking sequences. Thus, for decoding, UWD will use the output spike sequences to generate the noise-removed image. Based on our experiments, U-VTSNN is suitable for diverse popular datasets, and its computational efficiency is vastly superior to that of the same ANN architecture (over 274 times).

### 2.6. Loss function and backpropagation

#### 2.6.1. Rethinking spatio-temporal backpropagation (STBP)

A previous study has applied for training high-performance SNN (Wu et al., 2018; Jin et al., 2022). Noticeably, while examining the stability of a classification task, some researchers applied STBP for image generation (Comşa et al., 2021). The standard backpropagation only considers the spatial information, which can easily be underfitted and STBP overcomes that shortage. In order to compare STBP with our Independent-Temporal Backpropagation (ITBP) in the noise-removal task, the loss function corresponding to STBP is shown below.


(9) $${ℒ}_{STBP}=\frac{1}{N}\|y-\hat{y}{\|}_{F}^{2}$$

According to this loss function expression, the process of updating parameters is presented. To fairly compare, we show how $L_{STBP}$ updates $w_{n}^{j}$ in spatio-temporal domain. Other cases of updating parameters of STBP can be seen in Wu et al. (2018)'s work.


(10) $$\frac{\partial {ℒ}_{STBP}}{\partial w_{n}^{j}}=\sum_{t=1}^{T}\frac{\partial {ℒ}_{STℬP}}{\partial u_{t,n}^{i}}o_{t,n-1}^{j}$$

**Figure 4:**
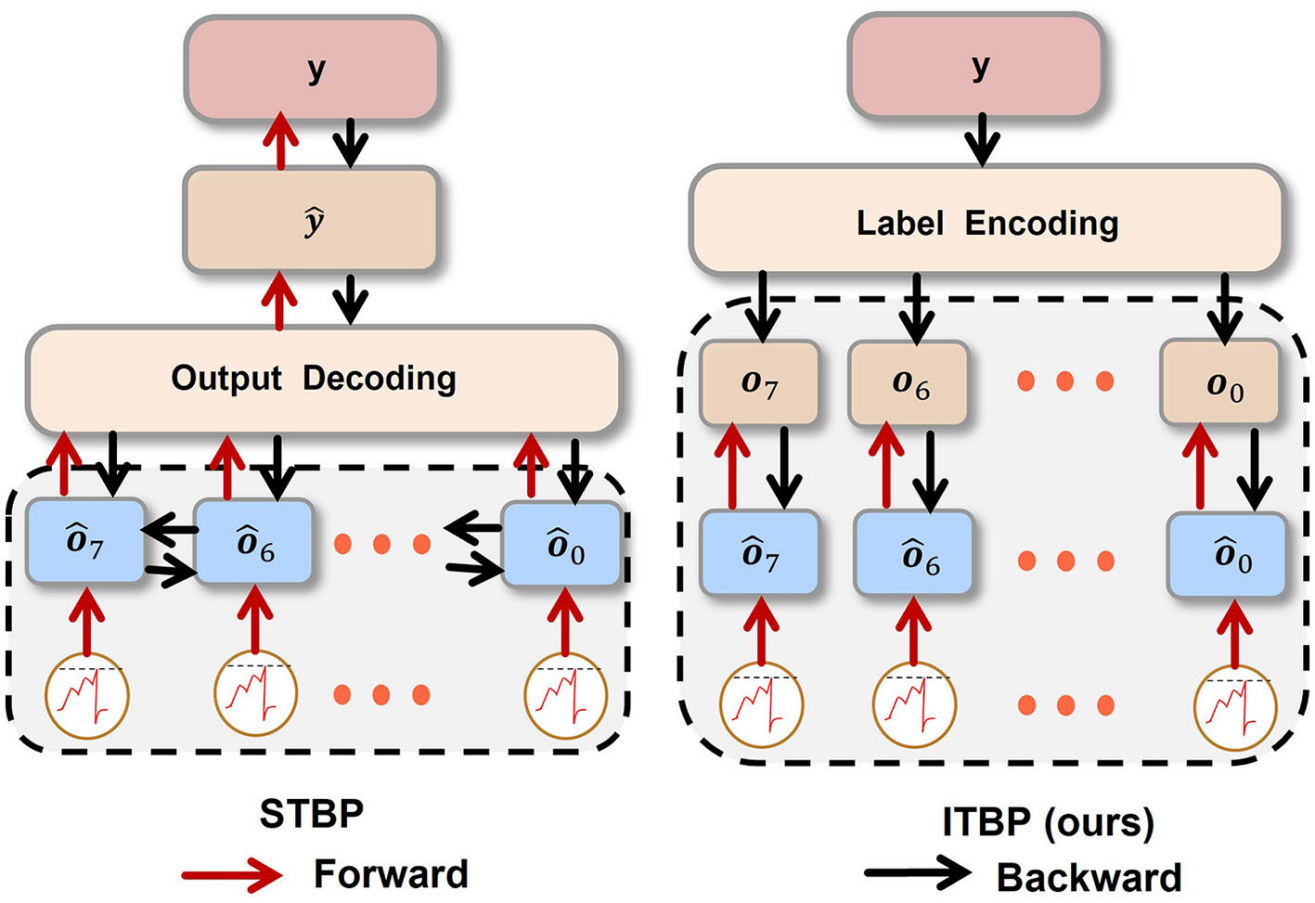
The procedure of STBP and ITBP. For STBP, the operated sequence {ô_7_, ô_6_, ⋯ , ô_0_} (we denote the sequence as ${\hat{\text{o}}}_{Seq}$) is transformed into ŷ via UWD. Then MSE between *y* and ŷ is calculated. For ITBP, *y* is transformed into input sequence {*o*_7_, *o*_6_, ⋯ , *o*_0_} (we denote the sequence as **o**_*Seq*_) by UWE. Then, calculate weighted MSE between ${\hat{\text{o}}}_{Seq}$ and **o**_*Seq*_ by Equation (11), where ${\hat{\text{o}}}_{Seq}$ is the operated sequence ready to be decoded.

As Equation (12) shown, while STBP updates $w_{n+1}^{j}$, $o_{t,n+1}^{i}$, $o_{t-1,n+1}^{i}$, and $o_{t,n}^{j}$ are all connected with $w_{n+1}^{j}$. In Figure 4, unlike STBP, error backpropagation of ITBP will not go through the decoder. Hence, ITBP is more efficient than STBP. Because error backpropagation of ITBP is in latent space but representational space for STBP. In other words, there is a risk of overfitting. Since there is a clear difference between ITBP and standard backpropagation: during the training process, ITBP only encodes the labels and does not decode network outputs; while ITBP does not encode the labels and does decode network outputs during the testing process. STBP has overcome standard backpropagation in noise removal task (Comşa et al., 2021). Later in the experiment, ITBP performed even better than STBP in a similar task.

#### 2.6.2. Independent-Temporal Backpropagation (ITBP)

To show the Independent-Temporal Backpropagation (ITBP) training framework, we create the loss function $L_{ITBP}$ where the weighted mean square error is used as the error index. The expression of it is described below:


(11) $${ℒ}_{ITBP}=\frac{1}{N}\sum_{t=0}^{T-1}2^{t}\|o_{t}-{\hat{o}}_{t}{\|}_{F}^{2}$$

Where *N* is the number of training examples and ||·||_*F*_ represents the Frobenius norm, *T* is the total time step and we set *T* = 8 for our UWE and UWD. From the equation above, we regard $L_{ITBP}$ as a function of *w* (weight). To obtain the derivative of $L_{ITBP}$ to *w* is necessary for the gradient descent. To obtain the final $\frac{\partial L_{ITBP}}{\partial w_{n}^{j}}$, the critical step is to obtain the $\frac{\partial L_{ITBP}}{\partial o_{t,n}^{i}}$ and $\frac{\partial L_{ITBP}}{\partial u_{t,n}^{i}}$ at time *t*. Now, we show the insight of getting the complete gradient descent. First, from Equations (2) to (**4**), the output of spiking neurons $o_{t,n+1}^{i}$ can be represented below:


(12) $$\begin{array}{l} o_{t,n+1}^{i}=h\left[u_{t-1,n+1}^{i}g\left(o_{t-1,n+1}^{i}\right)+\Sigma_{j}w_{n+1}^{j}o_{t,n}^{j}-V_{th}\right] \end{array}$$

Where $w_{n+1}^{j}$ is the synaptic weight which links the output of *n*+1 layer spiking neuron $o_{t,n+1}^{i}$ with the one of *n* layer $o_{t,n}^{j}$. According to Equations (2) to (**4**), we can calculate $\frac{\partial L_{ITBP}}{\partial o_{t,n}^{i}}$ and $\frac{\partial L_{ITBP}}{\partial u_{t,n}^{i}}$ as follows.


(13) $$\begin{array}{l} \frac{\partial L_{ITBP}}{\partial u_{t,n}^{i}}=\frac{\partial L_{ITBP}}{\partial o_{t,n}^{i}}\frac{\partial o_{t,n}^{i}}{\partial u_{t,n}^{i}} \end{array}$$

(14) $$\begin{array}{l} \frac{\partial L_{ITBP}}{\partial o_{t,n}^{i}}=\frac{\partial L_{ITBP}}{\partial o_{t,n+1}^{j}}\frac{\partial o_{t,n+1}^{j}}{\partial o_{t,n}^{i}} \end{array}$$

(15) $$\begin{array}{l} \frac{\partial o_{t,n+1}^{i}}{\partial o_{t,n}^{j}}=\frac{\partial o_{t,n+1}^{i}}{\partial u_{t,n+1}^{i}}\frac{\partial u_{t,n+1}^{i}}{\partial o_{t,n}^{j}}\\ \;=\sum_{j}\frac{\partial o_{t,n+1}^{i}}{\partial u_{t,n+1}^{i}}w_{n+1}^{j} \end{array}$$

By Equation (5), the following derivatives of surrogate function Equation (16) can be used for approximation.


(16) $$\begin{array}{l} \frac{\partial o_{t,n+1}^{i}}{\partial u_{t,n+1}^{i}}=\frac{1}{1+{\left(\pi x_{t+1,n}^{i}\right)}^{2}} \end{array}$$

Here, $\frac{\partial L_{ITBP}}{\partial u_{t,n}^{i}}$ is the intermediate variable on the step of updating parameters $w_{n}^{j}$, from Equations (14) to (16), we can solve Equation (13) as follows.


(17) $$\begin{array}{l} \begin{array}{c} \frac{\partial L_{ITBP}}{\partial u_{t,n}^{i}}={\left[\frac{1}{1+{\left(\pi x_{t+1,n}^{i}\right)}^{2}}\right]}^{2}\frac{\partial L_{ITBP}}{\partial o_{t,n+1}^{i}}\underset{j}{\sum }w_{n+1}^{j} \end{array} \end{array}$$

Hence, the way we update parameters will be shown below.


(18) $$\begin{array}{l} \frac{\partial {ℒ}_{ITBP}}{\partial w_{t,n}^{j}}=\frac{\partial {ℒ}_{ITBP}}{\partial u_{t,n}^{i}}\frac{\partial u_{t,n}^{i}}{\partial x_{t,n}^{i}}\frac{\partial x_{t,n}^{i}}{\partial w_{n}^{j}}\\ \;=\frac{\partial {ℒ}_{ITBP}}{\partial u_{t,n}^{i}}o_{t,n-1}^{j} \end{array}$$

To state how we update weights within one epoch clearly, Algorithm 2 is shown. For Independent-Temporal Backpropagation (ITBP) in this paper, it is a non-cross-path backpropagation. That means it only propagates spatially not temporally. In other words, it is a single-modal spatial representation which means single-modality simplifies and enhances ITBP's efficiency. Last but not least, ITBP only propagates spike sequences of coded labels.

**Algorithm 2:**
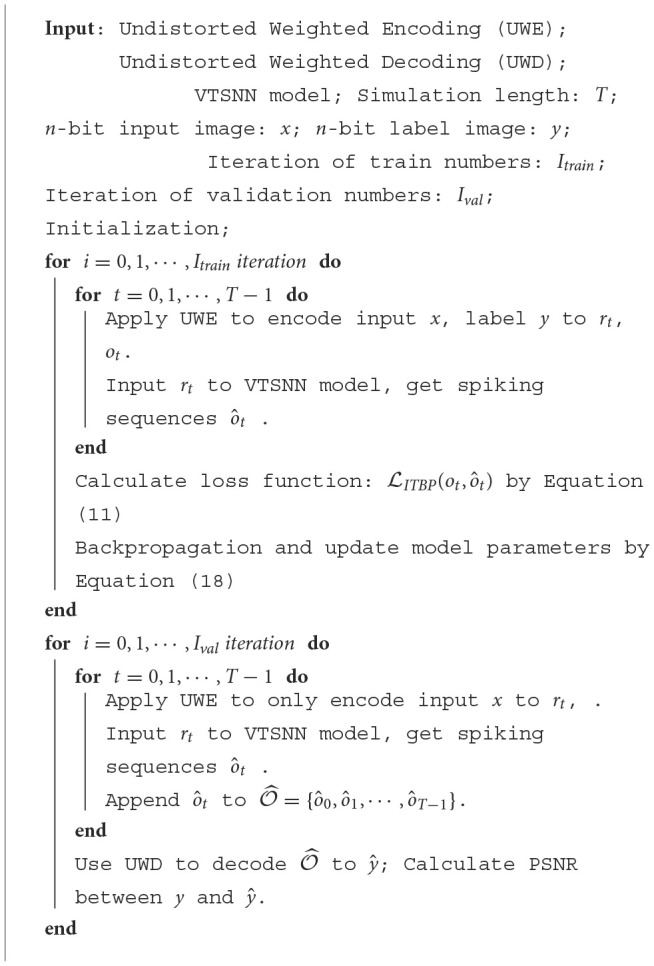
ITBP for one epoch.

## 3. Results

To demonstrate the superiority of our work and compare it to existing studies fairly, we choose widely used standard datasets for our experiments. Hence, we implemented VTSNN in PyTorch (Paszke et al., 2019), and evaluated it using MNIST (LeCun et al., 1998), F-MNIST (Xiao et al., 2017), and CIFAR10 (Krizhevsky et al., 2009). For MNIST and F-MNIST, we used 60,000 images for training and 10,000 images for evaluation which is the same as Comşa et al. (2021) in the noise-removal task. The input images were resized to 28 × 28. To expand the applicability of VTSNN, we also conducted experiments on CIFAR10. For CIFAR10, we used 50,000 images for training and 10,000 images for evaluation. The input images were resized to 32 × 32. Moreover, all noisy images used for training and testing contain Gaussian noise at each pixel, with η representing the noise variation in the image scale from 0 to 1. Moreover, our training details are as follows. On NVIDIA GeForce GTX 2080, the models are implemented using PyTorch. In addition, each layer's bias is set to False. The optimizer is Adam Optimizer, which updates the weight parameters of the network with the loss value for better gradient descent, and its initial learning rate is set to 0.001. Moreover, our batch size is 50 for both training and testing.

### 3.1. Comparison with existing works

**Figure 5:**
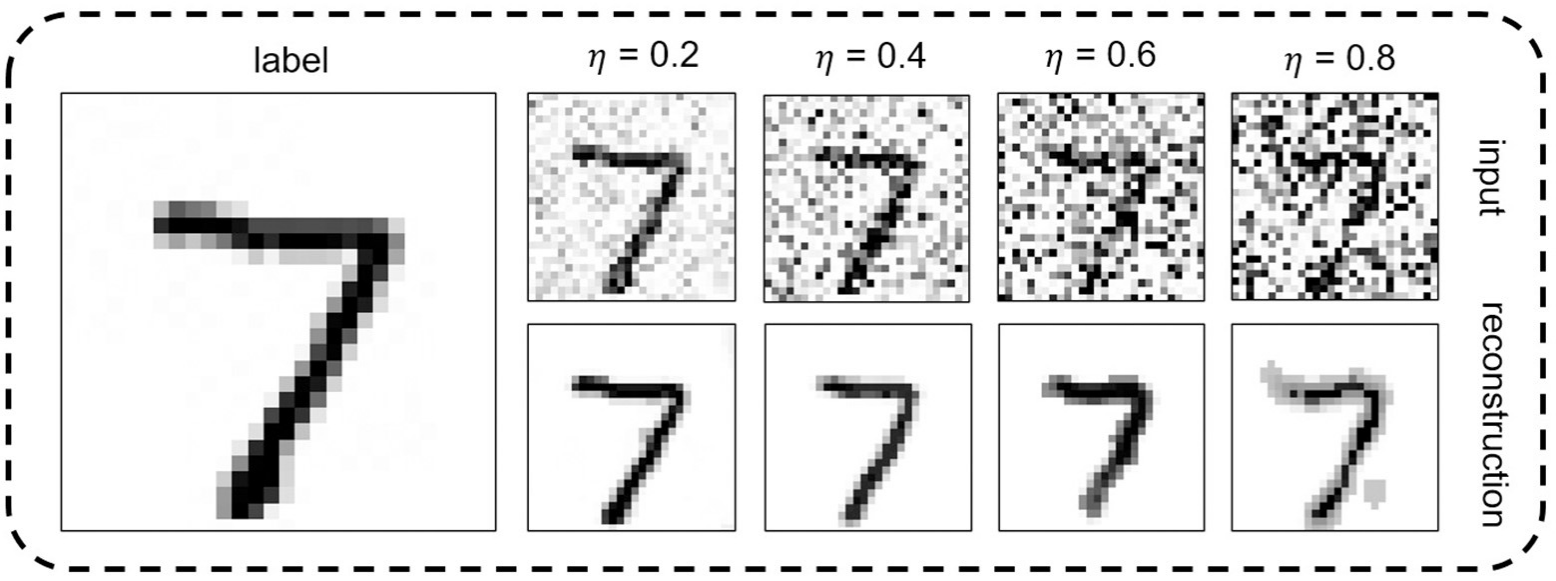
A digit from MNIST set is reconstructed by the proposed VTSNN incorporated into the commonly used U-net architecture and IF neuron, at different noise levels.

The performance of two VTSNN variants is compared with some models in Table 1. And a digit from MNIST dataset is reconstructed by our model is show in Figure 5. We train and test two variants based on the PyTorch framework, resulting in enhanced performance across all tasks. And, we compare the performance between ours and the methods proposed by Comşa et al. (2021) which is the only SNN-based image reconstruction attempt yet. On neuromorphically-encoded MNIST, the boost values of PSNR on four various noise levels are {4.26, 5.75, 7.03, 7.06} with only eight time steps. On neuromorphically-encoded F-MNIST, the boost value of PSNR on four various noise levels are {3.66, 4.09, 3.692, 3.93} with also eight time steps. Moreover, the value of PSNR on four various noise levels are {18.27, 14.08, 14.76, 13.16} on neuromorphically-encoded CIFAR10 with also eight time steps, which is quite competitive. Furthermore, our method can achieve higher performance in image reconstruction tasks by neuromorphic encoding/decoding circuits. Even compared with ANN-based work, on MNIST, at η=0.2, our VTSNN-IF performs superior to it. And the results are shown in Table 2.

**Table 1:** Comparison of PSNR on existing works for various noise levels and different datasets (Bold: the best).

**Method**	**MNIST**				**F-MNIST**				**CIFAR10**			
	η**= 0.2**	η**= 0.4**	η**= 0.6**	η**= 0.8**	η**= 0.2**	η**= 0.4**	η**= 0.6**	η**= 0.8**	η**= 0.2**	η**= 0.4**	η**= 0.6**	η**= 0.8**
SATC-16	17.06	16.60	15.24	14.49	17.54	16.76	16.04	15.61	–	–	–	–
SATC-32	19.11	17.40	16.06	15.10	18.01	17.17	16.72	15.90	–	–	–	–
**VTSNN-LIF**	22.99	21.74	19.04	19.05	20.85	20.82	19.86	18.71	15.85	10.53	10.96	9.47
**VTSNN-IF**	**23.57**	**23.15**	**23.09**	**22.56**	**21.67**	**21.26**	**20.64**	**19.83**	**18.27**	**14.08**	**14.76**	**13.16**

**Table 2:** Comparison of PSNR on MNIST at various noise level η = 0.2 for different encoding and decoding (Bold: the best) (Wu et al., 2018; Comşa et al., 2021; Kamata et al., 2022).

**Encoding**		**Decoding**		**Backpropagation**		**PSNR**
**UWE**	**TTFS**	**UWD**	**MPD**	**STBP**	**ITBP**	
✓	✓	✓	9.60
✓	✓	✓	8.00
✓	✓	✓	8.54
✓	✓	✓	9.76
✓	✓	✓	**23.57**
✓	✓	✓	11.91
✓	✓	✓	11.81
✓	✓	✓	20.70
Same architecture ANN						23.05

### 3.2. Ablation study

#### 3.2.1. Comparison between LIF neuron and IF neuron

Currently, research uses leaky-integrate-and-fire (LIF) neurons for SNN, believing its more complex differential equation (Gerstner et al., 2014) can boost performance. Our experiment disproves this bias. To conduct our experiment, we use commonly used datasets (MNIST, FMNIST, and CIFAR10). In our method, the parameters of the IF model are set as *V*_*reset*_ = None, *V*_*th*_ = 0.077 in 1 × 1 convolution layer (an experience parameter corresponds to best performance), and *V*_*th*_ = 1.0 in all the other convolution layers. In terms of LIF neurons, τ = 1.1, and all the other parameters are set identically to IF neurons. In the majority of instances, as shown in Table 1, IF neurons usually do better than LIF neurons at this task, regardless of the noise level or dataset.

#### 3.2.2. Comparison among different coding methods

TTFS and MPD are discussed relatively in depth in the rethinking part (Sections 2.3.1 and 2.4.1) and introduction. Since they have been used to generate images (Kamata et al., 2022). They are the two most comparable methods for our UWE and UWD. Table 2 displays all results. UWE is always superior to TTFS when conducting a univariate experiment, and UWD is always superior to MPD too. In addition, the UWE-UWD combination performs exceptionally well for STBP.

#### 3.2.3. Comparison between STBP and ITBP

Experiments demonstrate that ITBP is superior to STBP in terms of the PSNR evaluation metrics. $L_{STBP}$ and $L_{ITBP}$ are applied respectively with the same U-VTSNN architecture. Table 2 displays the outcomes of these two backpropagation techniques on the MNIST dataset with η = 0.2. All these results well proved the superiority of our ITBP.

## 4. Discussion

### 4.1. Classification for UWE

**Figure 6:**
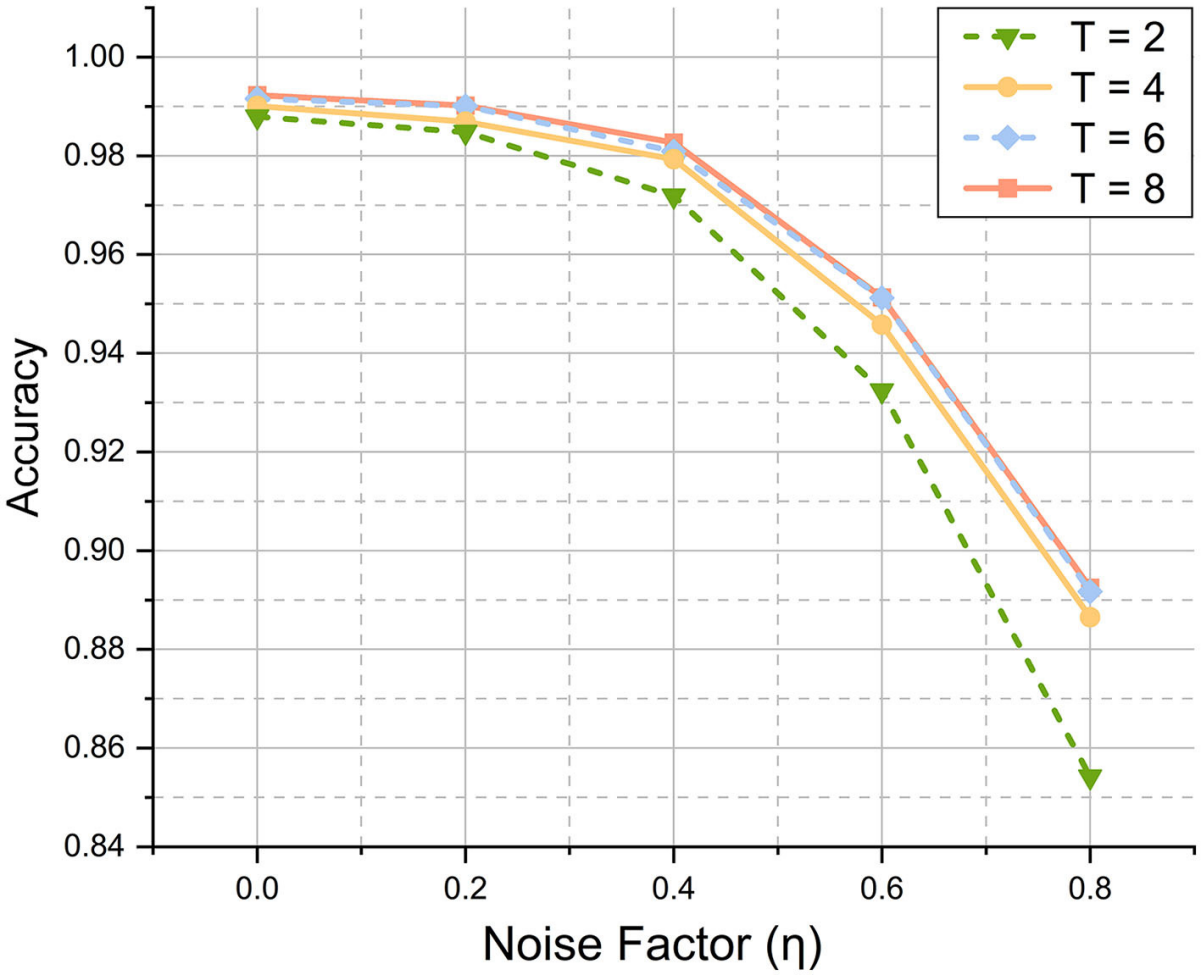
Results of classification task in MNIST dataset at different noise factors. For any *T*, while the noise level goes up the accuracy will decrease. However, even the worst case (*T* = 2, η = 0.8) will achieve a quite good result (85.2%). And the best case (*T* = 8, η = 0.0) can perform quite competitively (99.2%).

In addition to image reconstruction, VTSNN is capable of performing various tasks (Xu et al., 2021; Ran et al., 2022), such as medical detection (Ghosh-Dastidar and Adeli, 2009) and speech recognition (Mansouri-Benssassi and Ye, 2019). As mentioned in the Introduction, classification is a common assignment for SNN. To demonstrate the classification, we employ a VTSNN-based LeNet (VTLeNet) (LeCun et al., 1998) in which all activation functions are replaced by spiking neurons and UWE is used for encoding. Then, we employ VTLeNet to classify the MNIST dataset. Furthermore, varying levels of noise (η = 0.2, 0.4, 0.6, 0.8) are applied to the images in MNIST. Here, we are not attempting to attain optimal outcomes, but rather to test the stability of our UWE classification work. The results are presented as a line chart in Figure 6.

### 4.2. Energy consumption

In this section, we use the same network structure (Rathi and Roy, 2021; Zhu et al., 2022). Ideally, in the absence of spikes, no computations and active energy are used (Davies et al., 2018; Zhu et al., 2022). For the sake of fairness, we exclude convolutional computations for both and hold the above ideal conjecture. We traverse MNIST and count ANN activation function operations and SNN spikes. In these experiments, all spiking neurons are replaced with an ANN activation function (e.g., ReLU), and its total operations are counted.^2^ ANN then needs 18.39 M Flops^3^, while VTSNN needs 2.51 M FLOPS. In other words, #*OP*_*ANN*_=18.39 M, #*OP*_*SNN*_=2.51 M.

Following the practice (Zhu et al., 2022), in 45 nm CMOS, each ANN operation consumes 4.6 and 0.9 pJ for each spike (Horowitz, 2014). Thus, 32-bit ANN costs 18.39 M × 4.6 pJ = 8.46 × 10^−5^ J, or 273.77 times as much as 32-bit VTSNN. Moreover, details of how energy consumption is calculated can be found in Table 3. This method of calculation is generally accepted in the SNN field and we learned from Zhu et al. (2022). The ideal results are extremely encouraging and demonstrate SNN's immense potential. To realize these awe-inspiring effects, however, future research into hardware is required. The neuromorphic circuits in this paper may be a good harbinger.

**Table 3:** Comparison of energy based on the counting of operations between ANN and SNN.

**ANN**	**SNN**
Total params	0.12 M	0.12 M
(a) Spike rate	0	0.1366
^*a*^(b) #**OP*_*ANN*_*	18.39 M	0
^*b*^(c) #**OP*_*SNN*_*	0	2.51 M
^*c*^Energy(10^−7^J)	845.94	3.09
^*d*^ANN/SNN Energy	273.77	

^a^#*OP*_*ANN*_ is the total number of ANN operations if all spiking neurons are replaced with an ANN activation function (e.g., ReLU).

^b^#*OP*_*SNN*_ = *SpikeRate*× #*OP*_*ANN*_.

^c^Energy = #*OP*_*ANN*_× 4.6*pJ* + #*OP*_*SNN*_× 0.9*pJ*× *SpikeRate*.

^d^Each operation in ANN (SNN) consumes 4.6 pJ (0.9 pJ). ANN/SNN Energy can be calculated by $\frac{\left(b\right)\times 4.6}{\left(a\right)\times \left(c\right)\times 0.9}$.

### 4.3. Regularity of threshold voltage

**Figure 7:**
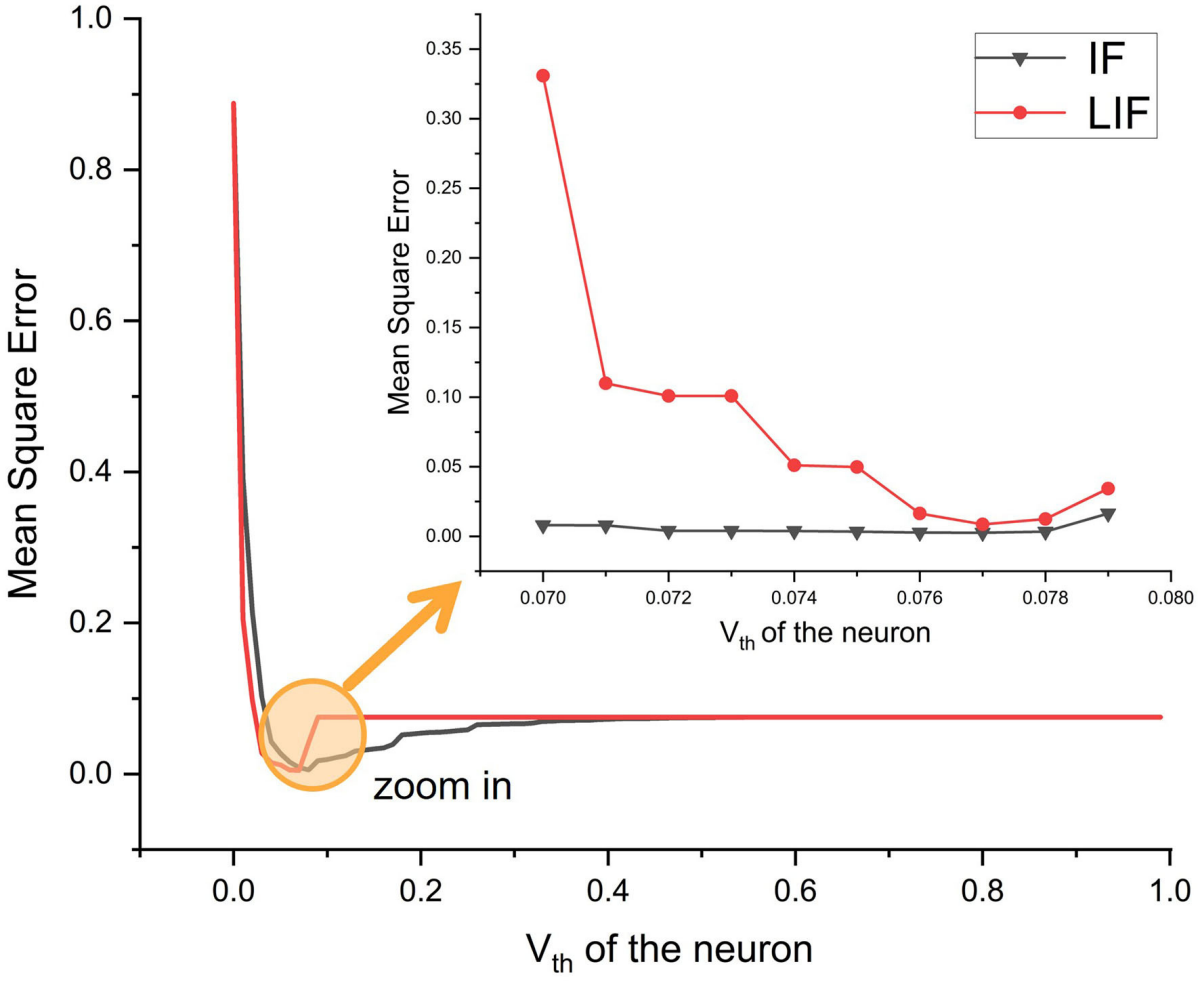
Performance of neurons in the final layer under various *V*_*th*_ conditions, with MSE as the evaluation metric. The circled and enlarged region illustrates the complexity of performance surrounding a specific *V*_*th*_ value (*V*_*th*_ = 0.1 here).

Experiments show *V*_*th*_ impacts outputs. To determine the regularity of that relation, we find the optimal *V*_*th*_ by attempts. Studies show a doubtful conjecture that increasing *V*_*th*_ increases spiking rate frequency, which improves performance (Niu et al., 2022). At each epoch, we count output spiking rate frequencies corresponding to different *V*_*th*_. Thus, we contradict that simple correlation. Figure 7 depicts the ebb and flow of performance regarding various *V*_*th*_. Recent studies about astrocytes harbor find during daytime and nighttime the threshold for the cell is different (Koronowski and Sassone-Corsi, 2021). This biological property inspired us. Other scholars in the SNN field also state that the dynamic membrane potential threshold, as one of the essential properties of a biological neuron is a spontaneous regulation mechanism that maintains neuronal homeostasis, i.e., the constant overall spiking firing rate of a neuron (Ding et al., 2022). Our discussion is motivated by the above biological research, and we hope to pique the interest of more academics to investigate the regularity of threshold voltage's insight.

### 4.4. Neuromorphic circuits

To enhance the efficacy of UWE and UWD, a simple neuromorphic circuit can be introduced. The UWE and UWD systems rely fundamentally on a binary encoding-decoding strategy. In particular, binary data is hardware-friendly, inspiring us to investigate ADC and DAC. The non-floating nature of the circuits embodies the spirit of neuromorphic chips and the successful avoidance of calculation through direct electronic responses.

**Figure 8:**
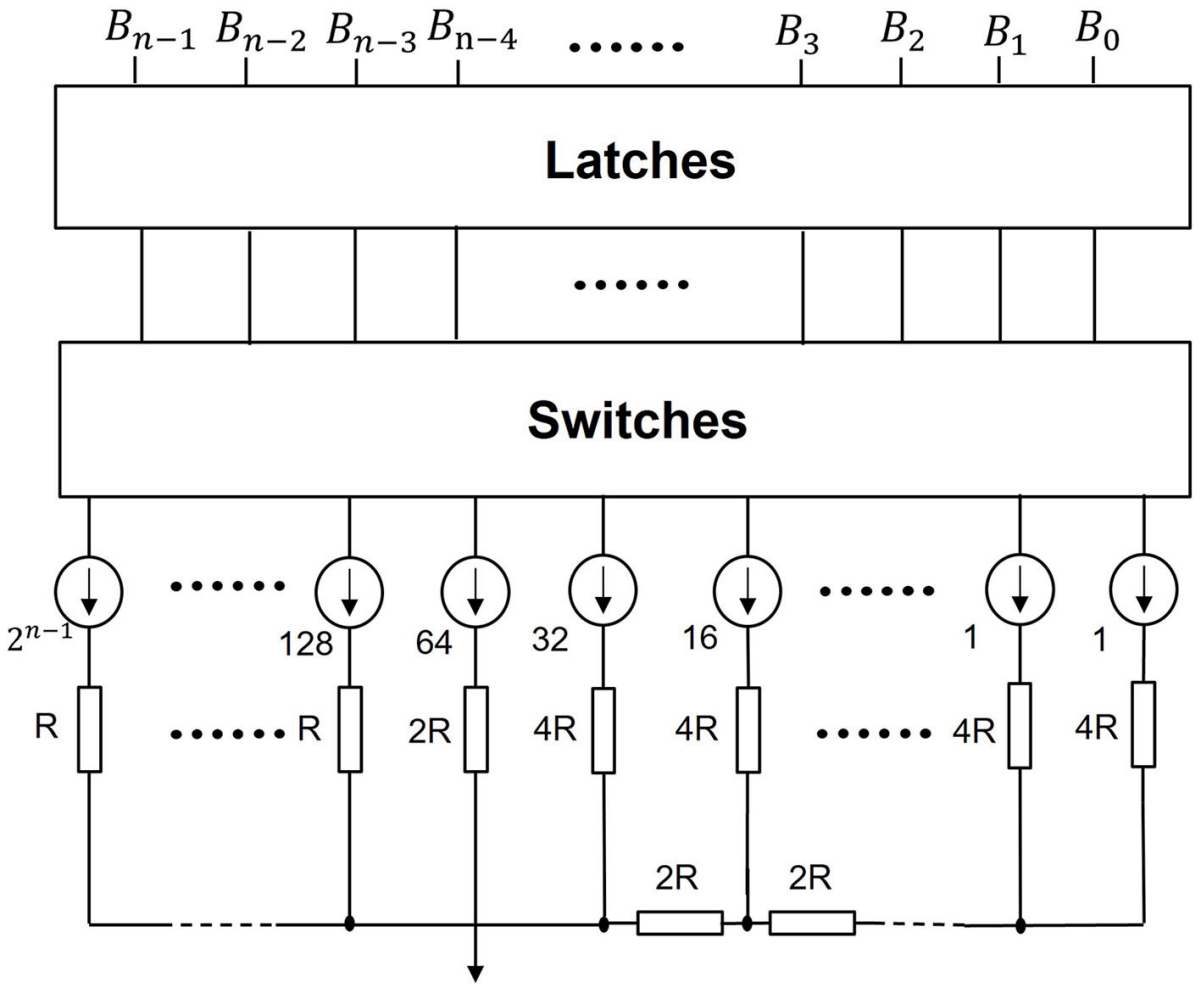
Neuromorphic decoding circuits. We use this simple neuromorphic DAC to realize our UWD. If a switch is on, the corresponding branch outputs 1. Otherwise, the branch outputs 0. This mechanism is designed to activate spikes. And with the resistors in series, the real pixel value is transferred.

As shown in Figure 8, UWD can be enabled by a simple DAC. Here, {*B*_0_, *B*_1_, ⋯ , *B*_*n*−1_} refers to spiking sequences of a pixel from networks. Whether a spike occurs depends on whether switches are on or off. The output of this neuromorphic chip is the real value of that pixel. Furthermore, the resistance network corresponds to *n*-bit decoding matrix *A* in Section 2.4.

**Figure 9:**
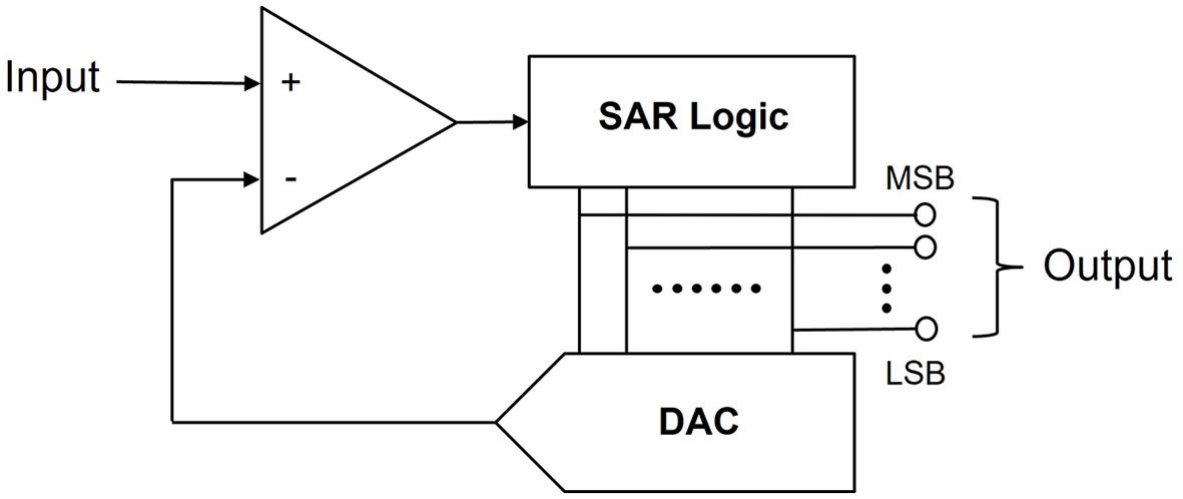
Neuromorphic encoding circuits. we use this simple neuromorphic SAR ADC to realize our UWE. Each real pixel value will be transferred into pixel spiking sequences.

Similarly, Figure 9 shows how to realize UWE without sample-hold circuits. A comparator is linked to SAR logic and the DAC model here is the circuits in Figure 8. Finally, MSB is the abbreviation of Most Significant Bit (*n*-bit) while LSB refers to Least Significant Bit (1-bit). This means MSB to LSB constitutes a binary sequence.

### 4.5. Limitation

The majority of direct training SNNs are currently trained with rather basic data. In addition, all of the current SNN-based image reconstruction research use very simple images (Comşa et al., 2021; Kamata et al., 2022). Similarly, our work here is unable to circumvent this difficulty. The reconstructed high-revolution images created by VTSNN are not optimal and seem blurry to the human eyes. In conclusion, SNN is still far behind ANN in image reconstruction tasks involving high-resolution images. However, SNN's potential cannot be ignored.

## 5. Conclusions

We have developed a novel spiking neuron network called VTSNN, where we adopt SNN with a virtual temporal dimension and a new backpropagation method. Besides, we raise Undistorted Weighted-Encoding to transfer the image into spiking information, which can be easily realized by a neuromorphic circuit to improve efficiency, as well as the symmetric process of Undistorted Weighted-Decoding. The experiments proved that VTSNN sometimes performs similarly to or better than ANN, for the same architecture and VTSNN is superior to all other comparable SNN models. Future research should focus on the development of hardware and the applicability of high-resolution images. There remain some constraints. The relationship between image low-level task performance and *V*_*th*_ is unclear. The proposed encoding-decoding circuits are not yet constructed physically.

## Data availability statement

The study's original contributions are given in the publication. And our code is available at this https://github.com/bollossom/VTSNN%20. For more information, please contact the relevant authors.

## Author contributions

X-RQ, Z-RW, and ZL designed and did the experiments, wrote the code, wrote the first draft of the manuscript, and contributed equally. R-JZ provided consultation on SNN knowledge, optimized the code, and helped with literature research. XW polished the draft manuscript and reviewed the code. M-LZ contributed to the concept and design. L-JD directed the projects and provided overall guidance. All authors contributed to the article and approved the submitted version.

## Footnotes

^1^In our work, SNN simulation length *T* = *n*, which means each bit is represented by a time step.

^2^The total number of ANN operations are counted by the torchstat package (Swall0w, 2018).

^3^In torchstat, the count operations are calculated by the formula (Molchanov et al., 2016) #*OP*_*ANN*_= $2HWC_{in}C_{out}K^{2}$ where *H* and *W* is the output feature map size; *C*_*in*_ is input channel; *K* is kernel size; *C*_*out*_ is the output channel.
